# The Autumn Society Meetings

**Published:** 1897-10

**Authors:** 


A Monthly Review of Medicine and Surgery.

*EDITORS:*

THOMAS LOTHROP, M. D. - - WM. WARREN POTTER, M. D.

All communications, whether of a literary or business nature, should be addressed to the managing editor: 284 FRANKLIN STREET, BUFFALO, N. Y.

VOL. XXXVII. OCTOBER, 1897. No. 3.

### THE AUTUMN SOCIETY MEETINGS.

THE series of medical society meetings scheduled for the autumn of 1897 is well under way and presents some features of unusual interest that command sufficient attention to justify comment at this time. The list was led by the American Association of Obstetricians and Gynecologists, that held its meeting at Niagara Falls, August 17–20, 1897, upon which editorial comment in detail was made in the September issue of the JOURNAL. This body held its meeting earlier than common this year for the purpose of enabling its members to enjoy the scenery at Niagara while it was at its best. We publish in this edition two excellent papers read at this meeting, as well as a synopsis of the proceedings, to which we invite the attention of our readers—all of which will; we believe, prove profitable reading.

Following close upon this meeting came that of the Twelfth International Congress, held at Moscow, August 19–26, 1897. Contrary to predictions in some quarters this congress appears to have been greatly successful from numerical, scientific and social viewpoints. About 100 Americans were present and Continental Europe was generously represented. Dr. J. B. Murphy, of Chicago, commanded the attention of the congress in the presentation of his work upon the anastomosis of arteries. This proved to be one of the most attractive features of the congress. The entertainments were keyed on a high pitch, ranging all the way from free railway passes to imperial hospitality. A number of the members of the congress were quartered at the Kremlin—a special courtesy accorded by the Czar of all the Russias. The *Medical Record* has published a full cable synopsis of the proceedings, which is a marked instance of journalistic enterprise as well as lavish expenditure. The congress will hold its next meeting at Paris in 1900.

The next organisation to hold forth was the British Medical Association, which held its sixty-fifth annual meeting at Montreal, August 31 to September 3, 1897, this being the first time in its history that it ever met outside of Great Britain. The attendance from the United States was about 300 invited guests, which was nearly one-half the aggregate number registered. Dr. T. G. Roddick, of Montreal, was the president, and it was largely through his efforts that the association came to Canada this year. The scientific work of the association does not appear to have been of the highest order—even the addresses were somewhat mediocre in character, if we except those of W. Japp Sinclair and Hermann M. Biggs. The distinguishing feature of the meeting was the presence of Lord Lister, whose genial good-nature and versatile talent made him the popular guest of honor at numerous social functions, one of which was given at Toronto on the evening of August 21, 1897. The medical profession there united in tendering a dinner to his lordship at the Toronto Club. Through the courtesy of the committee in charge a number of Americans were privileged to attend—an opportunity that was greatly appreciated by the guests from this side of the line. The becoming modesty with which Lord Lister on all occasions bore his blushing honors was the subject of general remark in approbation. The visit of Sir Joseph Lister to this country will long be remembered as one of the brightest professional epochs and it will serve to keep the Montreal meeting of the British Medical Association ever green in memory.

Turning now to the meetings to come we note that the Mississippi Valley Medical Association is announced to hold its annual meeting at Louisville, October 5–8, 1897. Efforts are making by the officers, with Dr. Thomas Hunt Stucky as president, and particularly by the committee of arrangements, to make this an especially interesting occasion. The railways have granted reduced rates, a large program containing many interesting titles by distinguished men is offered and the well-known hospitality of the city of Louisville, all conspire to justify the prediction made by the officers that this will be one of the most interesting medical meetings of the autumn season.

The Tri-state Medical Association—Alabama, Georgia and Tennessee—will hold its annual meeting at Nashville, October 12–14, 1897. The presiding officer, Dr. W. F. Westmoreland, of

Atlanta, is doing excellent work in organising this meeting, in which he is ably seconded by the secretary, Dr. Frank Trester-Smith. The preliminary program, already issued, bespeaks an occasion of great scientific interest. It must not be forgotten that the Tennessee Centennial Exposition is now in progress at Nashville.

The Central New York Medical Association, to be presided over by Dr. Edward B. Angell, of Rochester, will convene at Buffalo, October 19, 1897. Dr. Angell is well known to the readers of the JOURNAL and his enthusiastic interest in the welfare of the association will contribute to the success of this meeting. The profession of Western New York is cordially invited to attend. Dr. William C. Krauss, chairman of the committee of arrangements, states that he is assured that the attendance will be unusually large this year, that the program will be an interesting one and the meeting more than ordinarily successful.

The American Public Health Association will hold its twenty-fifth annual meeting at Philadelphia, October 26–29, 1897, under the presidency of Dr. Henry B. Horlbeck, of Charleston, S. C. The secretary, Dr. Irving A. Watson, of Concord, is busy with the affairs of the association and anticipates an unusually large gathering. The chairman of the committee of arrangements, Dr. Benjamin Lee, who is also secretary of the Pennsylvania state board of health, has made unusual preparations for both the social and scientific features of the meeting.

The Southern Surgical and Gynecological Association announces that its tenth annual meeting will be held at St. Louis, November 9, 10 and 11, 1897. This enterprising and progressive organisation includes some of the best medical talent of the South and always has instructive sessions characterised by scientific papers and spirited debates. The secretary, Dr. W. E. B. Davis, of Birmingham, who organised this association, is one of the most experienced medical society officials of the country as well as one of the most distinguished abdominal surgeons in the South. It is perfectly apparent that under his experienced management the forthcoming meeting of the association, to be presided over by Dr. George Ben Johnston, of Richmond, will be fully abreast of any previous meeting in the history of the organisation. Dr. Johnston's reputation, too, is national and his ability as a presiding officer is unexceled. The city of St. Louis is delightful during the Indian summer and everything seems to be conspiring in the interests of this meeting.

From the foregoing it will appear that there is a revival of interest in medical society affairs that bespeaks of the prosperity of the country in a most emphatic manner. When physicians attend medical societies in largely increasing numbers it is an index of financial ease that reflects beneficially upon other interests and occupations. Surely the effect of these autumn meetings will be advantageous to the general business interests of the country as well as to the physicians who attend them.

